# Direct action of FSH on testicular stem cells

**DOI:** 10.1186/s13287-019-1390-y

**Published:** 2019-08-23

**Authors:** Hiren Patel, Deepa Bhartiya

**Affiliations:** Stem Cell Biology Department, ICMR-National Institute for Research in Reproductive Health, Jehangir Merwanji Street, Parel, Mumbai, 400012 India

**Keywords:** Follicle-stimulating hormone, Testis, Stem cells, Very small embryonic-like stem cells, Spermatogonial stem cells

## Abstract

Recently published article by Pieri’s group suggested that follicle-stimulating hormone (FSH) activates canine testicular spermatogonial stem cells (SSCs) and results in increased expression of pluripotent markers and formation of germ cell clumps possibly via indirect paracrine effect of Sertoli cells. We disagree with their interpretations and herewith provide a better explanation to their findings. We have earlier reported the presence of pluripotent, very small embryonic-like stem cells (VSELs) as a sub-group among the SSCs in human and mouse testes and that both VSELs and SSCs express FSH receptors. Thus, FSH exerts a direct stimulatory action on the testicular stem/progenitor cells whereby VSELs undergo asymmetrical cell divisions to self-renew (result in upregulation of pluripotent markers) and give rise to slightly bigger SSCs which undergo symmetrical cell divisions and “clonal expansion” (rapid proliferation with incomplete cytokinesis) which was noted by the authors as “clump” formation. This action of FSH is mediated via alternately spliced FSHR3 rather than the canonical FSHR1 receptor isoform, and FSH exerts similar action on ovarian and uterine stem/progenitor cells also. Being quiescent by nature, VSELs survive chemotherapy. Transplanted germ cells colonize chemoablated tubules but do not differentiate into sperm since the testicular stem cell niche comprising Sertoli cells gets functionally compromised by chemotherapy. Transplanting healthy niche cells (Sertoli or bone marrow-derived mesenchymal cells) can restore spermatogenesis in chemoablated testes.

## Letter

Pieri et al. [1] have published an interesting study showing the effect of FSH on spermatogonial stem cell (SSC) expansion both in vitro and in vivo. Canine SSCs (cSSCs) increased in numbers in vitro in the presence of FSH confirmed by the increased numbers of GFRA1-positive cells and formation of germ cell clumps observed after 72 h. FSH treatment resulted in increased expression of early pluripotency marker OCT-4 and later germ cell markers PLZF, DAZL, c-Kit, and GFRA. On transplanting in chemoablated mouse testis, cSSCs were found to colonize and proliferate in the presence of FSH but there was no evidence of differentiation and increase in numbers of germ cells/sperm. This paracrine effect of FSH was discussed to be possibly via Sertoli cells which express follicle-stimulating hormone receptor (FSHR) and secrete more GDNF in the presence of FSH resulting in increased numbers of SSCs. They cited two of our articles as part of their methods to study GFP-positive SSCs.

The purpose to write this commentary is because major interpretations and conclusions reported by Pieri’s group need to be re-examined. Their findings are correct but still lack clarity on the role of FSH in adult testis that needs to be understood in proper perspective. We would like to discuss several facets of our findings to provide a novel understanding of the role of FSH on adult testicular stem cells.

1. *Testis harbors OCT-4-positive pluripotent stem cells in addition to SSCs*: We have reported that both adult human and mouse testicular stem cells include a sub-group of pluripotent stem cells termed very small embryonic-like stem cells (VSELs) along with SSCs [2–4]. VSELs were recently reviewed [2, 5] and are developmentally linked to primordial germ cells that possibly survive in adult tissues in small numbers. VSELs express pluripotent markers including nuclear OCT-4A whereas the SSCs express cytoplasmic OCT-4B. Besides testis, VSELs exist in all adult organs including the ovary and uterus. Two distinct populations of stem cells exist in adult tissues including pluripotent VSELs and slightly bigger tissue-specific progenitor ovarian stem cells (OSCs) in the ovary [6] and endometrial stem cells (EnSCs) in the uterus [7] similar to SSCs in the testis [4].
2. *Effect of FSH on stem cells in adult mammalian testis*: FSH treatment upregulated pluripotent markers in the testis. We have reported an increase in pluripotent OCT-4-positive stem cells after FSH treatment in mouse testis, ovaries, and uterus [2–4, 7]. In addition to OCT-4, other embryonic markers including Sox2 and Nanog also show increased expression and comprise essential transcription factors required to maintain the pluripotent VSELs phenotype in adult tissues.
3. *FSH stimulates SSCs to expand and form clumps or “colonies”*: FSH activated the SSCs to undergo proliferation resulting in a clump formation, with paracrine influence by the Sertoli cells as suggested by the authors. Rather, the testicular stem cells including VSELs and SSCs express FSHR, and FSH has a direct effect as reported for the first time in the literature by our group for both the testis [4] and ovary [8]. We have also shown that this effect of FSH is possibly mediated via alternately spliced FSHR3 and not via canonical FSHR1. The clumps reported by the authors represent clonal expansion of SSCs. Similarly, FSH treatment results in the formation of germ cell nests when ovarian stem cells are cultured in the presence of FSH [8]. Similar clumps were also reported in mouse uterus, and Western blotting showed the presence of FSHR isoforms in mouse uterus [7]. Pluripotent VSELs undergo asymmetrical cell divisions (ACD) to self-renew and give rise to SSCs/OSCs/EnSCs which in turn undergo rapid expansion, symmetrical cell divisions, and clonal expansion [4, 8, 9].
4. *Transplanted SSCs colonize and expand in response to FSH but fail to differentiate in chemoablated mouse testis*: This needs to be clearly understood. We have reported that VSELs survive chemotherapy whereas the Sertoli cells get functionally compromised. The authors observed long-term survival and colonization of cSSCs in chemoablated mouse testis, but they did not differentiate since Sertoli cells become non-functional as a result of chemotherapy. Transplantation of mesenchymal cells directly in the chemoablated testis (not intratubular route through the rete testis) helped to achieve differentiation since they provided an improved paracrine support to the surviving VSELs to restore spermatogenesis [3, 10].
5. Both bone marrow and testicular VSELs when cultured on a Sertoli cell bed and in the presence of Sertoli cell-conditioned medium differentiate into germ cells and sperm [2].

To conclude, FSH action is not limited to only the ovary and testis, and besides Sertoli and granulosa cells, it exerts a direct action on the stem cells to undergo self-renewal and clonal expansion. Alternately spliced FSH receptor isoform biology needs to be studied in greater details, and one needs to acknowledge both the stem cells and their niche to ensure regeneration of chemoablated testis.

## Data Availability

NA

## Abbreviations

FSH: Follicle-stimulating hormone
FSHR: Follicle-stimulating hormone receptors
VSELs: Very small embryonic-like stem cells
SSCs: Spermatogonial stem cells
OSCs: Ovarian stem cells
EnSCs: Endometrial stem cells
